# Editorial: New discoveries and challenges in pediatric infectious diseases: epidemiological, clinical, and pathogenic advances

**DOI:** 10.3389/fped.2026.1904336

**Published:** 2026-07-03

**Authors:** Dechuan Kong, Jiehao Cai

**Affiliations:** 1 Department of Communicable Diseases Control and Prevention, Shanghai Municipal Center for Disease Control and Prevention, Shanghai, China; 2 Children’s Hospital of Fudan University, Shanghai, China

**Keywords:** emerging and re-emerging pathogens, epidemiology, pathogenic mechanisms, pediatric infectious diseases, post-pandemic

## 1 Background and scope

Pediatric infectious diseases remain a major global health challenge, threatening children's physical health, education, and family well-being, while imposing a substantial socioeconomic burden. Before the COVID-19 pandemic, the resurgence of scarlet fever caused by Group A *Streptococcus* (1–4) and measles outbreaks (5–9) had already drawn worldwide concern. After the pandemic, unusual seasonal rebounds of respiratory syncytial virus(RSV) and influenza (10–17), as well as outbreaks of unexplained pediatric hepatitis (18–20), have further underscored the urgent need for updated epidemiological, clinical, and pathogenetic research.

This Research Topic, *New Discoveries and Challenges in Pediatric Infectious Diseases: Epidemiological, Clinical, and Pathogenic Advances*, published in *Frontiers in Pediatrics*, aimed to capture the latest advances across this multidisciplinary field. The 36 articles collected here attracted researchers from around the world, including China, Italy, Norway, Egypt, Qatar, Brazil, Kuwait, and other countries, reflecting truly global participation. The 36 articles collected here cover emerging and re-emerging infections, novel diagnostic technologies, clinical management strategies, antimicrobial resistance, and host immunity. As of the time of this editorial, the Research Topic has achieved 40k Topic views, 31k Article views, and 6,306 Article downloads. Below we highlight key contributions across these domains.

## 2 Epidemiological insights into changing disease patterns

Several studies provided crucial updates on the epidemiology of common and re-emerging pediatric infections. Tong et al. examined long-term epidemiological patterns of pediatric infectious diseases in an urban district of Shanghai from 2015 to 2023, revealing how the pandemic reshaped disease circulation over nearly a decade. Zhang et al. analyzed influenza A, influenza B, and *Mycoplasma pneumoniae* infections in hospitalized children with acute respiratory tract infection (ARTI) in Xi'an from 2021 to 2023, revealing co-circulation patterns and post-pandemic shifts. Similarly, Duan et al. reported on the epidemiology of *Chlamydia pneumoniae* infection in children with ARTI in Chengdu during 2022–2023, filling a key data gap for this often-overlooked pathogen. For hand, foot, and mouth disease (HFMD), Li et al. examined 11-year epidemiological features (2014–2024) in a Gansu Province city, identifying long-term trends and seasonal patterns.

Beyond common respiratory viruses, Abughosh et al. provided insights into the epidemiological and clinical burden of bronchiolitis among hospitalized children, issuing a call for preventive interventions. Zheng et al. assessed the impact of demographic, clinical, and viral factors on severe influenza in young children in Fuzhou, offering region-specific risk stratification. For surveillance infrastructure, van den Berg et al. conducted a pilot study in the Netherlands to improve infectious disease surveillance in child daycare settings, a critical environment for transmission. These epidemiological studies provide essential baseline data for public health planning and early warning systems.

## 3 Emerging and Re-emerging pathogens: unusual and life-threatening infections

The case reports in this Research Topic highlighted rare but severe pediatric infections that demand clinical awareness. Fu et al. described a case of primary amoebic meningoencephalitis caused by *Naegleria fowleri* in a 6-year-old girl—a nearly always fatal infection requiring rapid diagnosis. Li et al. reported a case of *Rickettsia felis* meningoencephalitis in a child, accompanied by a literature review, emphasizing the need to consider vector-borne pathogens in unexplained neurological illness. Hua et al. presented the successful management of scrub typhus-associated hemophagocytic lymphohistiocytosis (HLH) with doxycycline and immunomodulation, offering a treatment template for this severe complication. Additionally, Qing et al. described a case of X-linked severe combined immunodeficiency (SCID) complicated by *Talaromyces marneffei* infection caused by a novel pathogenic gamma-chain gene of the interleukin-2 receptor (*IL2RG*) mutation, illustrating how primary immunodeficiencies can unmask rare opportunistic infections. These reports expanded the known spectrum of pediatric infectious diseases and provided practical management guidance.

## 4 Clinical management, diagnostic innovations, and antimicrobial resistance

Optimizing clinical care was a central theme. Peng et al. compared differences in onset and clinical manifestations of *Mycoplasma pneumoniae* pneumonia between children and adults during the post-COVID-19 era, while identifying factors influencing severe cases—providing age-stratified insights for clinical decision-making. Zhu et al. screened early predictive serum biomarkers for refractory *Mycoplasma pneumoniae* infection in children and constructed a combined predictive model, a valuable tool for early identification of severe cases.

For critical pertussis, Alghounaim et al. conducted a multicenter survey of pediatric intensivists in the Arabian Gulf Cooperation Council region, revealing significant practice variability in intensive care management of pertussis—highlighting an urgent need for standardized protocols. Han et al. retrospectively analyzed clinical features and risk factors for outcomes of postoperative bacterial meningitis in pediatric neurosurgery patients from 2013 to 2023, offering evidence-based guidance for this high-risk population.

Chipol-Ceja et al. explored SARS-CoV-2 reinfection as a possible associated factor for long COVID in children and adolescents, adding to our understanding of post-viral sequelae in the pediatric population. The neonatal period received special attention in a review by Zheng et al. on the neonatal lung microbiome, which discusses how this dynamic determinant influences respiratory health, disease susceptibility, and potential novel therapeutics.

## 5 Pathogenic mechanisms and host immunity

Mechanistic insights were essential for developing vaccines and immunomodulatory therapies. Kong et al. argued in a mini-review that the era of “Infectious Diseases+” has arrived, advocating for multi-disciplinary integration (including immunology, genomics, and data science) in pediatric infectious disease prevention and control. This perspective aligned with the detailed case report by Qing et al., where a novel *IL2RG* mutation led to combined immunodeficiency and opportunistic infection—demonstrating the power of genetic diagnosis to explain host susceptibility.

## 6 Future directions and challenges

Despite the progress reflected in these 36 articles, major challenges remain. The practice variability in pertussis management identified by Alghounaim et al. called for international guidelines. The long-term epidemiological shifts documented by Tong et al. reminded us that post-pandemic disease patterns may take years to stabilize. The rare but devastating infections highlighted in case reports remind us that many pediatric infectious diseases lack rapid diagnostics and evidence-based treatments. Furthermore, as Kong et al. emphasize, breaking down disciplinary silos and integrating infectious diseases with public health, genomics, and data science will be crucial for future preparedness.

We hope this Research Topic serves as a valuable resource for clinicians, epidemiologists, and basic scientists working to protect children from infectious diseases. By sharing the discoveries and openly discussing current challenges, we aimed to accelerate progress toward better prevention, diagnosis, and treatment for children worldwide.

## References

[1] Liu Y Chan TC Yap LW Luo Y Xu W Qin S. Resurgence of scarlet fever in China: a 13-year population-based surveillance study. Lancet Infect Dis. (2018) 18(8):903–12. 10.1016/S1473-3099(18)30231-7 29858148 PMC7185785

[2] Andrey DO Posfay-Barbe KM. Re-emergence of scarlet fever: old players return? Expert Rev Anti Infect Ther. (2016) 14(8):687–9. 10.1080/14787210.2016.1195684 27249582

[3] Yung CF Thoon KC. A 12 year outbreak of scarlet fever in Singapore. Lancet Infect Dis. (2018) 18(9):942. 10.1016/S1473-3099(18)30464-X 30152353

[4] Lamagni T Guy R Chand M Henderson KL Chalker V Lewis J. Resurgence of scarlet fever in England, 2014–16: a population-based surveillance study. Lancet Infect Dis. (2018) 18(2):180–7. 10.1016/S1473-3099(17)30693-X 29191628

[5] Zipprich J Winter K Hacker J Xia D Watt J Harriman K. Measles outbreak–California, December 2014-February 2015. MMWR Morb Mortal Wkly Rep. (2015) 64(6):153–4. Erratum in: *MMWR Morb Mortal Wkly Rep*. (2015) 64(7):196. PMID: 25695321; PMCID: PMC4584705 25695321 PMC4584705

[6] Kakoullis L Sampsonas F Giannopoulou E Kalogeropoulou C Papachristodoulou E Tsiamita M. Measles-associated pneumonia and hepatitis during the measles outbreak of 2018. Int J Clin Pract. (2020) 74(2):e13430. 10.1111/ijcp.13430 31573732

[7] Slade TA Klekamp B Rico E Mejia-Echeverry A. Measles outbreak in an unvaccinated family and a possibly associated international traveler—orange county, Florida, December 2012-January 2013. MMWR Morb Mortal Wkly Rep. (2014) 63(36):781–4.PMID: 25211542; PMCID: PMC4584693. 25211542 PMC4584693

[8] Doll MK Correira JW. Revisiting the 2014–15 disneyland measles outbreak and its influence on pediatric vaccinations. Hum Vaccin Immunother. (2021) 17(11):4210–5. 10.1080/21645515.2021.1972707 34495822 PMC8828106

[9] Parpia AS Skrip LA Nsoesie EO Ngwa MC Abah Abah AS Galvani AP. Spatio-temporal dynamics of measles outbreaks in Cameroon. Ann Epidemiol. (2020) 42:64–72.e3. 10.1016/j.annepidem.2019.10.007 31902625 PMC7056523

[10] Hu H. Post-pandemic shifts in the age distribution of RSV, influenza A, and rhinovirus infections: a four-year pediatric surveillance study. BMC Public Health. Published online June 3. (2026). 10.1186/s12889-026-28011-x PMID: 42231216 PMC13445722 42231216

[11] Alzahrani AJ El-Shahidy S Bizri NE Fakhr AE Sheikh AE Mahmoud A. Respiratory pathogen epidemiology in the post-pandemic era: a multicenter retrospective study from Saudi Arabia. BMC Infect Dis. Published online May. (2026) 26. 10.1186/s12879-026-13645-4 PMID: 42192365 PMC13393929 42192365

[12] García-García ML Alonso-López P Alcolea S María A Iglesias-Caballero M Sánchez-León R. Clinical and epidemiological changes in severe viral respiratory infections in pediatric patients after the COVID-19 pandemic in Spain. Sci Rep. Published online May 18. (2026). 10.1038/s41598-026-52362-5 PMID: 42151312 PMC13376559 42151312

[13] Dewey G Meyer AG Garcia RG Santillana M. Uncovering the post-pandemic timing of influenza, RSV, and COVID-19 driving seasonal influenza-like illness in the United States: a retrospective ecological study. Lancet Reg Health Am. (2026) 55:101359. 10.1016/j.lana.2025.101359 41551297 PMC12804126

[14] Munro APS House T. Cycles of susceptibility: immunity debt explains altered infectious disease dynamics post-pandemic. Clin Infect Dis. (2026) 81(6):1173–6. 10.1093/cid/ciae493 39390969

[15] Park M Choi WS Cowling BJ. Shifts in influenza and respiratory syncytial virus infection patterns in Korea after the COVID-19 pandemic resulting from immunity debt: retrospective observational study. JMIR Public Health Surveill. (2025) 11:e68058. 10.2196/68058 40700345 PMC12309781

[16] Lee PI Hsueh PR Chuang JH Liu MT. Changing epidemic patterns of infectious diseases during and after COVID-19 pandemic in Taiwan. J Microbiol Immunol Infect. (2024) 57(5):685–90. 10.1016/j.jmii.2024.07.002 39048396

[17] Perroud AP Coronel DL Rivas E. Analyzing seasonal trends of respiratory syncytial virus circulation using Latin American national surveillance database during pre- and post-COVID-19 pandemic. Int J Infect Dis. (2025) 161:108095. 10.1016/j.ijid.2025.108095 41043634

[18] Morfopoulou S Buddle S Torres Montaguth OE Atkinson L Guerra-Assunção JA Moradi Marjaneh M. Genomic investigations of unexplained acute hepatitis in children. Nature. (2023) 617(7961):564–73. 10.1038/s41586-023-06003-w 36996872 PMC10170458

[19] Shteyer E Mor O Waisbourd-Zinman O Mozer-Glazberg Y Arnon R Hecht Sagie L. The outbreak of unexplained acute hepatitis in children: the role of viral infections in view of the COVID-19 pandemic. Viruses. (2024) 16(5):808. 10.3390/v16050808 38793689 PMC11125843

[20] Mandal S Simmons R Ireland G Charlett A Desai M Coughlan L. Paediatric acute hepatitis of unknown aetiology: a national investigation and adenoviraemia case-control study in the UK. Lancet Child Adolesc Health. (2023) 7(11):786–96. 10.1016/S2352-4642(23)00215-8 37774733

